# *Cryptococcus neoformans* and *Cryptococcus gattii* Species Complexes in Latin America: A Map of Molecular Types, Genotypic Diversity, and Antifungal Susceptibility as Reported by the Latin American Cryptococcal Study Group

**DOI:** 10.3390/jof7040282

**Published:** 2021-04-09

**Authors:** Carolina Firacative, Wieland Meyer, Elizabeth Castañeda

**Affiliations:** 1 Studies in Translational Microbiology and Emerging Diseases (MICROS) Research Group, School of Medicine and Health Sciences, Universidad del Rosario, Bogota 111221, Colombia; 2 Molecular Mycology Research Laboratory, Centre for Infectious Diseases and Microbiology, Research and Education Network Westmead Hospital, Faculty of Medicine and Health, Sydney Medical School-Westmead Clinical School, Marie Bashir Institute for Infectious Diseases and Biosecurity, Westmead Institute for Medical Research, The University of Sydney, Sydney 2145, Australia; wieland.meyer@sydney.edu.au; 3 Grupo de Microbiología, Instituto Nacional de Salud, Bogota 111321, Colombia; ecastaneda21@gmail.com

**Keywords:** cryptococcosis, Latin America, molecular types, *Cryptococcus*, antifungal susceptibility, MLST

## Abstract

Cryptococcosis, a potentially fatal mycosis, is caused by members of the *Cryptococcus neoformans* and *Cryptococcus gattii* species complexes. In Latin America, cryptococcal meningitis is still an important health threat with a significant clinical burden. Analysis of publicly available molecular data from 5686 clinical, environmental, and veterinary cryptococcal isolates from member countries of the Latin American Cryptococcal Study Group showed that, as worldwide, *C. neoformans* molecular type VNI is the most common cause of cryptococcosis (76.01%) in HIV-infected people, followed by *C. gattii* molecular type VGII (12.37%), affecting mostly otherwise healthy hosts. These two molecular types also predominate in the environment (68.60% for VNI and 20.70% for VGII). Among the scarce number of veterinary cases, VGII is the predominant molecular type (73.68%). Multilocus sequence typing analysis showed that, in Latin America, the *C. neoformans* population is less diverse than the *C. gattii* population (*D* of 0.7104 vs. 0.9755). Analysis of antifungal susceptibility data showed the presence of non-wild-type VNI, VGI, VGII, and VGIII isolates in the region. Overall, the data presented herein summarize the progress that has been made towards the molecular epidemiology of cryptococcal isolates in Latin America, contributing to the characterization of the genetic diversity and antifungal susceptibility of these globally spreading pathogenic yeasts.

## 1. Introduction

In Latin America, as is the case globally, cryptococcosis affects predominantly immunocompromised male patients, with HIV infection being the main predisposing risk factor [1]. Cryptococcal meningitis, which is the main presentation of cryptococcosis, remains a significant burden in adults from many regions of the world where there is a high HIV seroprevalence [2]. Despite great improvements in antiretroviral therapy, cryptococcosis is still associated with high morbidity and mortality [1,3]. In Latin America, 3.7 million people lives with HIV, of which ~40% do not have access to antiretroviral therapy. In 2019 alone, there were 120,000 new HIV infections, representing an increase of 7% of new diagnoses annually compared with 2010 [4]. Although there are few reports regarding the incidence and prevalence of cryptococcosis in the region, studies from Brazil and Colombia have reported an average annual incidence from 2.4 to 4.5 cases of meningeal cryptococcosis per million inhabitants, in the general population, and 3000 to 3300 cases per million HIV-infected people [5,6]. In reports from Argentina, Colombia, Guatemala, Mexico, and Venezuela, cryptococcosis has had a prevalence from 10 to 76% among the most frequent mycosis [7,8,9,10,11,12]. In addition, the mortality ranges between 30 and 60% of cases, although, in Brazil, it has been reported to be as high as 73% [13]. Together, these data show that cryptococcosis is still an important health threat in Latin America.

For the last few decades, several widely used molecular methods have been applied worldwide to determine the genotypes of clinical, environmental, and veterinary isolates of the members of the *Cryptococcus neoformans sensu lato* and *Cryptococcus gattii sensu lato* species complexes, the etiological agents of cryptococcosis, to study their geographical distribution, molecular epidemiology, and population genetics, which have contributed to a deeper understanding of these deadly pathogens. Among such molecular techniques, PCR fingerprinting [14], amplified fragment length polymorphisms (AFLP) [15,16], microsatellite typing [17], restriction fragment length polymorphisms (RFLP) [18], and multilocus sequence typing (MLST) [19] have allowed for the identification of eight major molecular types within the species complexes globally: VNI (AFLP1) and VNII (AFLP1A/B) for *C. neoformans* var. *grubii* serotype A isolates; VNIV (AFLP2) for *C. neoformans* var. *neoformans* serotype D isolates; VNIII (AFLP3) for hybrids between the serotypes A and D; and VGI (AFLP4), VGII (AFLP6), VGIII (AFLP5), and VGIV (AFLP7) for *C. gattii* serotypes B and C isolates. More recently, additional molecular types, represented by fewer isolates, have been described in more restricted geographical regions, such as the molecular type VNB in *C. neoformans*, which was initially reported in Botswana [20], and the molecular type VGV in *C. gattii*, reported among environmental isolates in the Central Zambezian Miombo Woodlands [21]. Lately, whole genome sequencing (WGS) [22,23], a more recently developed and increasingly more affordable molecular technique, has been applied not only to identify major molecular types, but also to provide information about the genomic diversity between the genotypes of both *C. neoformans* and *C. gattii* species complexes, providing a much higher resolution than MLST, and serving as a basis for population genetics studies rather than simple strain typing. Although, in 2015, there was a proposal for naming seven separate species among the cryptococcal molecular types, named *C. neoformans* for VNI, VNII, and VNB; *C. deneoformans* for VNIV; *C. gattii* for VGI; *C. deuterogattii* for VGII; *C. bacillisporus* for VGIII; *C. tetragattii* for VGIV; and *C. decagattii* for VGIV and VGIIIc/AFLP10 [24], the terms “*C. neoformans* species complex” and “*C. gattii* species complex” will be used through this review, as a consensus on the nomenclature of the cryptococcal species has not yet been achieved [25] and most reviewed publications do not utilize the seven-species name system, but rather the molecular types.

The above-mentioned molecular methods have been essential tools to study the *C. neoformans*/*C. gattii* species complexes, giving foundations to better understand the differences in the geographical and environmental distribution, the host preference, virulence, clinical manifestations, and antifungal susceptibility of the isolates between major molecular types/species [26,27,28,29,30]. The aim of this review was thus to gather and analyze publicly available data on the molecular epidemiology of *C. neoformans* and *C. gattii* species complexes in Latin America, in order to enable a more complete picture of the distribution of the molecular types in the region, their diversity, and their antifungal susceptibility profiles, which in turn will contribute to the comprehension of the global structure of these pathogens.

## 2. Materials and Methods

Data were obtained from the previous revision on cryptococcosis in Latin America, published by two authors of the current review, covering publications dating from 1999 to mid-2017 [1]. In addition, a new literature search was undertaken comprising studies published from mid-2017 to December 2020, focusing on the molecular epidemiology of *Cryptococcus* and cryptococcosis in Latin America, including publications in English and Spanish from PubMed, SciELO, and Google databases. The literature search was based on the keywords “cryptococcus”, “cryptococcosis”, “molecular type”, “PCR fingerprinting”, “AFLP”, “microsatellite typing”, “RFLP”, “MLST”, “WGS”, or “antifungal susceptibility”, in combination with the names of 20 Latin American countries, which participate in the Latin American Cryptococcal Study Group (Argentina, Bolivia, Brazil, Chile, Colombia, Costa Rica, Cuba, Dominican Republic, Ecuador, El Salvador, Guatemala, Haiti, Honduras, Mexico, Nicaragua, Panama, Paraguay, Peru, Uruguay, and Venezuela). In addition, researchers from the Latin American Cryptococcal Study Group were contacted to request data from studies that have not been included in the above-mentioned public databases. All publications, in which the major molecular type, independently of the technique used, and/or data on MLST of the isolates were provided, were included in the review.

Data obtained from MLST typing, performed using the International Society of Human and Animal Mycology (ISHAM) consensus MLST scheme for *C. neoformans* and *C. gattii*, which includes six genetic loci, *CAP59*, *GPD1*, *LAC1*, *PLB1*, *SOD1*, and *URA5* genes, and the intergenic spacer region IGS1 [19], were gathered. Based on the obtained sequence data, a dendrogram, per species complex, showing the genetic relationship between the isolates of *C. neoformans* and *C. gattii* from Latin America was constructed with the program MEGA 7.0 [31], using maximum likelihood analysis of the seven concatenated loci. To estimate the genetic diversity of the *C. neoformans* and *C. gattii* populations, the Simpsons diversity index (*D*) was calculated per species complex, as well as for the source of the isolates, considering the number of sequence types (STs) found and the frequency of each ST. The range of *D* is from 0 to 1, where high scores (close to 1) indicate high diversity and low scores (close to 0) indicate low diversity [32].

Data on antifungal susceptibility testing were also gathered and analyzed. Independently of the methodology applied, values of the range and the geometric mean of minimal inhibitory concentrations (MICs) for amphotericin B, 5-fluorocytosine, fluconazole, itraconazole, voriconazole, and/or posaconazole were considered for the analysis, when these values were specified per molecular type. As there are no clinical breakpoints for *Cryptococcus* spp., the epidemiological cut-off value (ECV) >95% per molecular type and per antifungal drug was assessed, when available, to define if the isolates are distributed or not among the wild-type population [27,28] (Table S1).

Most antifungal susceptibility data included in this review were obtained using the microdilution method in RPMI broth according to the M27-A3 guideline of the Clinical and Laboratory Standards Institute (CLSI) [33]. However, data obtained by other methods, such as the European Committee on Antimicrobial Susceptibility Testing-Subcommittee on Antifungal Susceptibility Testing (EUCAST-AFST) method E.Def.7.2. [34], E-test strips (bioMerieux, Marcy l’Étoile, France), the Casitone broth microdilution method [35], Sensititre YeastOne plates (Thermo Scientific, Waltham, MA, USA), the Vitek-2 Compact system (bioMerieux, France), and disk diffusion (Cecon-Sensifungidisc, São Paulo, Brazil), have also been reported.

## 3. Results

### 3.1. C. neoformans VNI and C. gattii VGII Predominate in Latin America

To date, 106 publications have reported the major molecular type of 5686 isolates of *C. neoformans* and *C. gattii* from Latin America, identified mostly by RFLP (57.14%). From those, 65 publications were retrieved from the revision on cryptococcosis in Latin America and 41 were found afterwards [15,17,18,20,32,35,36,37,38,39,40,41,42,43,44,45,46,47,48,49,50,51,52,53,54,55,56,57,58,59,60,61,62,63,64,65,66,67,68,69,70,71,72,73,74,75,76,77,78,79,80,81,82,83,84,85,86,87,88,89,90,91,92,93,94,95,96,97,98,99,100,101,102,103,104,105,106,107,108,109,110,111,112,113,114,115,116,117,118,119,120,121,122,123,124,125,126,127,128,129,130,131,132,133,134,135]. Most publications were exclusive for one country; however, some other publications reported data from two or more countries. Overall, molecular type data were available from 15 of the 20 Latin American countries (Table 1). No molecular data of cryptococcal strains from the Dominican Republic, El Salvador, Haiti, Nicaragua, and Panama have yet been reported.

From the isolates, dating back to 1961 [18], 3976 were recovered from clinical specimens (69.93%), followed by 1691 environmental samples (29.74%) and 19 veterinary (0.33%) cases. From the clinical isolates, it was determined that the molecular type VNI, corresponding to *C. neoformans* var. *grubii*, serotype A, predominantly causes cryptococcosis in Latin America (76.01%), followed by *C. gattii* molecular type VGII (12.37%). In the environment, although with slightly different proportions, the molecular types VNI (68.60%) and VGII (20.70%) also predominate, with different reservoirs for each species complex. While *C. neoformans* species complex is mostly recovered from avian excreta, decaying organic matter, and soil, *C. gattii* species complex is associated with diverse species of both native and foreign trees.

Clinical case reports were uncommon, comprising only 16 publications, describing four VGI, five VGII, and three VGIII *C. gattii* cases, as well as four VNI *C. neoformans* cases. From them, cryptococcosis occurred rarely in HIV-infected people, but rather in patients with other risk factors, such as renal transplant and non-Hodgkin’s lymphoma, as well as in immunocompetent hosts, including children. Apart from meningitis, bone marrow involvement, primary cutaneous cryptococcosis, and a tongue lesion were also reported [48,59,60,72,74,86,91,101,108,115,117,118,124,131,132,134].

Together with a review of cryptococcosis in children from Colombia, where the authors reported the molecular type of the isolates (29 VNI, 3 VNII, and 2 VGII) [97], one study from Brazil reported six apparently healthy children (up to 12 years old) and seven adolescents/young adults aged 13 to 19 years presenting with cryptococcal meningitis by *C. gattii* VGII, from which five cases (38.4%) were fatal [51]. In addition to those studies, three case reports, caused by *C. gattii*, have been published in Latin America with the molecular data of this mycosis in pediatric patients. One VGII case of a 5-year-old boy from Brazil [48], one VGIII case of a 7-year-old girl from Argentina [117], both with successful outcomes, and one fatal VGII case of a 10-year-old boy from Colombia [108]. Remarkably, the cases from Argentina and Colombia were from indigenous, otherwise healthy kids, who had central nervous system involvement.

From the less frequently recovered veterinary isolates, reported in two articles and eight case reports from Brazil, Cuba, and Chile, the molecular type VGII was predominant (73.68%), followed by VNI (15.79%), VGIII (5.26%), and VGI (5.26%) (Table 1). When stated, veterinary cases were from seven cats, five dogs, two goats, a guinea pig, and a cheetah held in a zoo [55,62,70,71,76,81,123,133].

**Figure 1:**
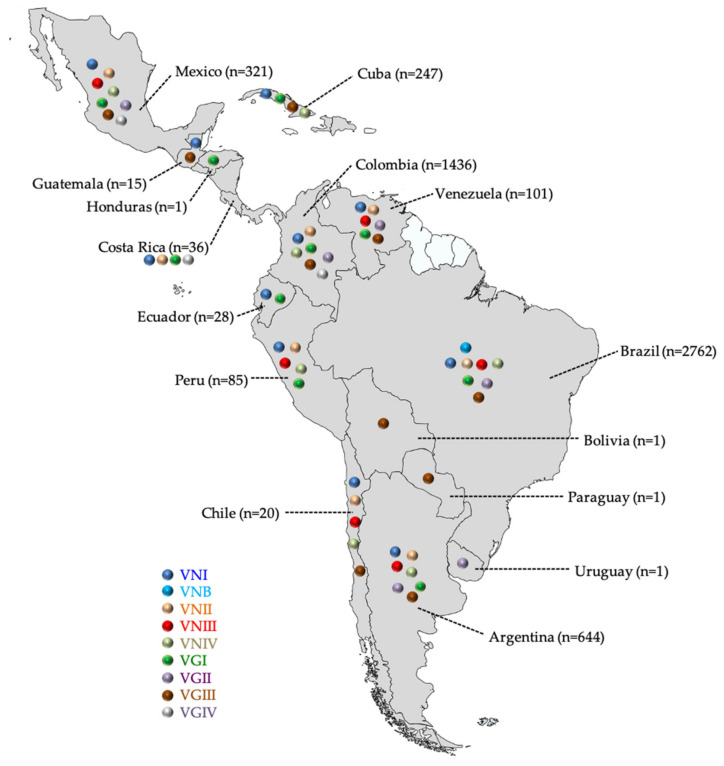
Geographic distribution of the molecular types of *Cryptococcus neoformans* and *Cryptococcus gattii* species complexes in Latin America.

The combined analysis of the molecular data also showed that only in Mexico have all eight major molecular types been reported, while in Brazil and Argentina, there are no reports of VGIV isolates, and in Colombia, VNIII has not been reported (Figure 1). However, in Brazil, 13 VNB isolates, previously thought to be exclusive from Botswana, have been reported not only from the environment [75], but also from clinical cases [85]. Also in Brazil, a new singular cluster, denominated as AFLP1C, representing a unique molecular type, was identified in 23 isolates [77]. In addition, apart from VNIII, which is the most common hybrid found in *C. neoformans*, other inter- and intra-specific hybrids have been reported in Latin America. Fifteen isolates described as VNII/VNIV hybrids were reported from Argentina, another isolate with the same genotype VNII/VNIV, two isolates VNI/VGII, and one isolate VNI/VNII from Brazil, as well as five isolates VNI/VNII and one isolate VNI/VGII from Colombia [85,119,136,137].

When stated, mating type alpha was the most common mating type in both *C. neoformans* and *C. gattii* isolates from Latin America. In the region, mating type **a** has been reported in *C. neoformans* in only two clinical VNI isolates from Brazil [53]. In contrast, in *C. gattii*, mating type **a** is slightly more common. In Colombia, 15 environmental VGII isolates, 1 VGI, and 12 VGII clinical isolates were mating type **a** [94,95,106], as well as 17 clinical and 3 environmental VGII isolates from Brazil [61,68,77], as well as 2 VGIII clinical isolates recovered in Mexico [32].

### 3.2. In Latin America, C. neoformans Species Complex Is Genetically Less Diverse than C. gattii Species Complex

Taking advantage of the data analysis, unique nomenclature, and interlaboratory comparability of MLST, it was possible to gather 367 MLST profiles of *C. neoformans* and 400 of *C. gattii* isolates from Latin America, reported in 26 publications [20,32,61,66,68,73,75,79,81,84,87,90,91,98,100,106,109,117,123,124,128,138,139,140,141,142]. MLST studies with clinical and environmental *C. neoformans* isolates have been reported from Brazil (77.11%), Peru (12.53%), Colombia (9.54), and Argentina (0.82%), while MLST studies with clinical, environmental, and veterinary *C. gattii* isolates have been reported from Brazil (65.25%), Colombia (26.25%), Mexico (5%), Argentina (1%), Venezuela (1%), and Uruguay (0.75%), as well as Cuba, Guatemala, and Paraguay with one isolate each (0.25%).

From the MLST profiles, the isolates were more frequently VNI (93.73%) among *C. neoformans* and more frequently VGII (74.75%) among *C. gattii*. While the number of isolates of *C. neoformans* and *C. gattii* studied so far in Latin America by MLST differs only slightly (367 vs. 400), there is a considerable difference in the number of identified STs (41 vs. 149). Thus, the genetic diversity, as calculated with the Simpsons diversity index (*D*), is clearly lower in *C. neoformans* compared with *C. gattii* (0.7104 vs. 0.9755). In addition, when analyzing the diversity of the isolates in both populations, depending on the source, it is evident that, in *C. neoformans*, the environmental isolates are more diverse than the clinical isolates, while in *C. gattii*, not only the clinical, but also the veterinary isolates are more diverse than those recovered from the environment (Table 2).

**Figure 2:**
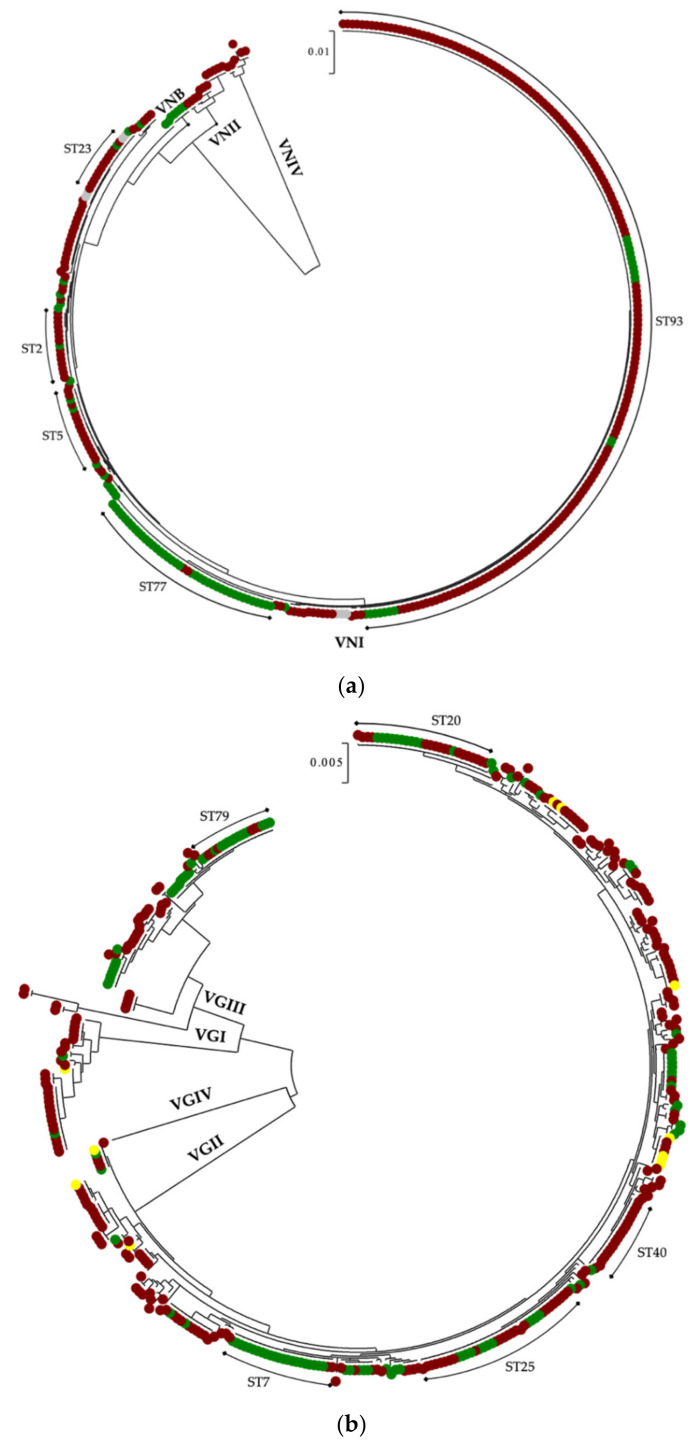
Dendrograms showing the genetic relationship of clinical (red), environmental (green), and veterinary (yellow) (**a**) *Cryptococcus neoformans* species complex (*n* = 367) and (**b**) *Cryptococcus gattii* species complex (*n* = 400) isolates from Latin America, according to multilocus sequence typing (MLST) data. The five most common sequence types, ST93, ST77, ST2, ST5, and ST23 for *C. neoformans*, and ST25, ST20, ST7, ST79, and ST40 for *C. gattii*, are indicated in each dendrogram. Isolates from an unknown source are indicated in grey.

From the 41 STs identified in *C. neoformans* species complex, 5 STs contained more than 75% of the isolates, which included both clinical and environmental samples. ST93 was the most frequent ST (52.04%), followed by ST77 (11.17%), ST2 (4.90%), ST5 (4.63%), and ST23 (3%) (Figure 2a). In contrast, from the 149 STs identified in *C. gattii* species complex, the 5 more frequent STs contained only 30.5% of the isolates. ST25 was the most common ST (9%), followed by ST20 (6.5%), ST7 (6%), ST79 (5.25%), and ST40 (3.75%). While ST25, ST20, ST7, and ST79 grouped clinical and environmental isolates, ST40 grouped only clinical isolates (Figure 2b). Of note, ST20 and ST7 correspond to the sub-types VGIIa and VGIIb, respectively, which were responsible for the Vancouver Island outbreak, which started in 1999 and is still ongoing [143]. In 11 veterinary cases caused by *C. gattii*, 8 STs were identified. From those, ST185 has been identified as well in human and environmental samples, ST182, ST248, and ST309 in human samples, but ST198, ST442, ST486, and ST489 are, so far, exclusively veterinary.

### 3.3. Most Isolates of C. neoformans and C. gattii Species Complexes from Latin America Show a Wild-Type Antifungal Susceptibility; However, Non-Wild-Type VNI, VGI, VGII, and VGIII Isolates Have Also Been Identified

The susceptibility to the commonly used antifungal drugs has been determined in 570 cryptococcal isolates from Latin America, based on data gathered from 23 publications [17,32,52,54,58,59,60,63,65,66,72,77,79,81,85,89,90,91,116,117,123,124,134]. From the 570 isolates, all have data on antifungal susceptibility to fluconazole, 434 to amphotericin-B, 296 to itraconazole, 251 to voriconazole, 250 to 5-fluorocytosine, and 192 to posaconazole. In order of frequency, the isolates were VNI (58.42%), VGII (19.47%), VGIII (10%), VNIV (6.32%), VNII (2.81%), VGI (1.23), VNB (1.05%), and VNIII (0.70%) (Table 3 and Table 4), recovered mainly from Brazil (65.79%), followed by Argentina (14.56%); Cuba (10%); Colombia (6.49%); Mexico (2.46%); and Bolivia, Guatemala, Paraguay, and Venezuela with one isolate each (0.18%).

Although most cryptococcal isolates in Latin America are wild-type to all antifungal drugs, in *C. neoformans*, among the molecular type VNI, non-wild-type isolates to amphotericin-B [58,89,116] and fluconazole [58,63,65,72,85,89,116] have been reported in Brazil and Argentina, to itraconazole in Brazil and Cuba [17,65], and to voriconazole in Brazil [89]. From the fluconazole non-wild-type VNI isolates, one was a case recovered from an HIV-positive patient with relapsing/refractory cryptococcosis that became fluconazole non-susceptible after 26 months (MIC from 16 to 32 µg/mL) and long-term use of liposomal amphotericin-B [72]. Moreover, among the VNI isolates, even though the MIC was not reported, resistance to amphotericin-B and to itraconazole was found in three and six isolates, respectively, belonging to the ST93, the most common MLST genotype in Latin America [75]. By disk diffusion, five and one VNI isolates from Brazil were reported to be intermediate (I) and resistant (R) to fluconazole, respectively (inhibition haloes diameters of I: 19–14 mm and R: <14 mm) [52].

Similarly, in *C. gattii*, non-wild-type VGII isolates to fluconazole [54,85], itraconazole [81], and voriconazole [81,89] have been reported in Brazil. Among the molecular type VGIII, fluconazole non-wild-type isolates have been described in Argentina, Colombia, Cuba, and Mexico [32,117,134]. In addition, although there are no data on ECV for amphotericin-B in VGIII, one isolate from Colombia and one from Mexico, of this molecular type, have shown high MICs to this antifungal drug (2 and 1 µg/mL, respectively) [32]. In one clinical case from Brazil [60] and one veterinary case from Cuba [123], both caused by *C. gattii* molecular type VGI, 5-fluorocytosine non-wild-type isolates were reported as well. By disk diffusion, two VGII isolates from Brazil were reported to be resistant to fluconazole (inhibition halo diameter <14 mm) [52].

## 4. Discussion

Analysis of more than 5000 cryptococcal isolates recovered in Latin American countries, which have data on the major molecular type, allowed to establish a precise description of the molecular epidemiology of the *C. neoformans*/*C. gattii* species complexes in the region, together with the genetic diversity and antifungal susceptibility of the isolates. Regardless of the country, in Latin America, *C. neoformans* VNI is not only the main etiologic agent of cryptococcosis (~76%) but is also the most recovered molecular type from environmental reservoirs (~69%), as it occurs globally [26]. Cryptococcosis by *C. gattii*, which is significantly less frequent in the world (<20%) [26], is also less frequent in Latin America, and it is most commonly caused by the molecular type VGII (~13%), which differs from other regions, as VGI isolates prevail in Asia, Europe, and Oceania, and VGIV in Africa [30]. Although VGII also predominates among the *C. gattii* isolates causing disease in North America, as well as in the environment, its prevalence refers mostly to the VGIIa subtype, which is influenced by the great effort made to understand the cause of the Vancouver Island outbreak and its clonal expansion into the USA [144,145].

In Latin America, the incidence of cryptococcal meningitis by *C. neoformans*, which mirrors the high number of HIV infections in the region, is not reducing because there is still a significant percentage of patients with no access to antiretroviral therapy or, despite availability, there may be problems of adherence and retention in HIV care, as reported elsewhere [2]. The number of people with HIV/AIDS who are undiagnosed, lost to follow-up, and living in resource-limited areas leads in addition to a late or incorrect diagnosis, which worsens prognosis and increases mortality [1]. Cryptococcosis by *C. gattii*, on the other hand, is also an important public health problem in Latin America, as it affects mostly HIV-seronegative individuals, apparently immunocompetent and, although rare, children. Notably, pediatric cryptococcosis has been reported in 32 and 29% of all cases in certain areas of Brazil and Colombia, respectively, where there is a high prevalence of *C. gattii* VGII infection in immunocompetent patients, which represents a unique prevalence pattern of this mycosis in children in the world [51,96,97,146,147,148]. Together, this reaffirms the endemic occurrence of primary cryptococcosis and early cryptococcal infection in some Latin American countries, although genetic determinants, which can be risk factors for the affected patients, have not been fully studied.

In the environment in Latin America, *C. neoformans* prevails in soil enriched with excreta, mostly from pigeons and other birds, such as captive birds, as reported globally [26]. However, after the first report on the isolation of *C. gattii* from the Australian native tree *Eucalyptus camaldulensis* [149], both cryptococcal species, although mostly *C. gattii,* have been successfully recovered from detritus, plant material, tree hollows, decayed woods, and even houses built with wood of a large variety of trees in Latin America, including native trees such as *Acacia visco*, *Cassia grandis*, *Hymenaea courbaril*, *Moquilea tomentosa*, *Peltophorum dubium, Plathymenia reticulata*, *Tabebuia avellanedae*, *Tabebuia guayacan*, *Tabebuia rosea*, *Tipuana tipu*, and *Roystonea regia*, as well as introduced trees such as *Casuarina equisetifolia*, *Cedrus deodara*, *Corymbia ficifolia, Croton* spp., *Cupressus sempervirens*, *Delonix regia*, *E. camaldulensis*, *Eucalyptus tereticornis*, *Eucalyptus* spp., *Ficus* spp., *Grevillea robusta*, *Phoenix* spp., *Senna siamea*, *Terminalia catappa*, and *Ulmus campestris*, among others [1,150,151,152]. Remarkably, as Latin American countries have a wide diversity of climates, landscapes, and ecosystems, both *C. neoformans* and *C. gattii* have been recovered from rainforest to desertic and dry environments, from urban and rural cities to native forest, and from sea-level to altitudes exceeding 2700 m [152,153,154]. Knowing the saprophytic source of cryptococcal species, the most recovered molecular types from clinical samples, VNI and VGII, also prevail in the environment in Latin America.

Even though cryptococcosis can occur in a very wide range of terrestrial and marine animals, including domestic, free-living, and wildlife, veterinary cases are more likely to go unrecognized, undiagnosed, and unreported compared with clinical cases [155]. In a greater extent than human cryptococcosis, most countries lack reliable information on the prevalence and incidence of animal cryptococcosis, which is not a reportable disease; hence, epidemiological investigations are further hampered. Yet, despite the few reports of cryptococcal infection in animals in Latin America, it is of note that *C. gattii* VGII is reported more frequently than *C. neoformans* VNI causing veterinary cases, which is opposite to the prevalence of these two molecular types in human and environmental samples. However, owing to the small number of isolates analyzed, it is not possible to make assumptions about this discrepancy. As in clinical cases, a correlation between veterinary cases and the geographical distribution of *C. neoformans* and *C. gattii* has been reported in Australia [156], Vancouver Island (Canada) [143,145], and the USA [157,158]. The occurrence of veterinary cases in Latin America should be nevertheless emphasized, because, while cryptococcosis is not transmissible between animals or humans, animals act as crucial sentinels for human cryptococcal infection [155].

In Latin America, except for certain cases in Colombia, Brazil, and Mexico, clinical, environmental, and veterinary cryptococcal isolates are almost always mating type alpha, regardless of species complex and molecular type, which obeys the distribution of mating types globally [26,30]. The rare finding of mating type **a** in *C. neoformans* in Latin America may explain the extensive clonality, low diversity (*D* = 0.7104), and overrepresentation of certain genotypes (STs) in this population, as it may not be sexually recombining in the region. The presence of both mating types in a population, which may lead to sexual reproduction, has been described, for instance, in the highly diverse *C. neoformans* population in Africa, where 22% of the VNB and 1 to 4% of the VNI isolates are mating type **a**, reproduce sexually in the laboratory, and generate fertile progeny [73,159,160]. Similarly, the higher frequency of mating type **a** *C. gattii* isolates in Latin America may explain the higher diversity of this population (*D* = 0.9755) and may indicate that there is genetic recombination, which has already been described in the VGII and VGIII populations by MLST and WGS studies [32,68,135]. Sexual reproduction of cryptococcal species in the environment, however, remains a mystery.

The analysis of the antifungal susceptibility profiles of members of the *C. neoformans*/*C. gattii* species complexes in Latin America showed that most VNI, VGI, VGII, and VGIII isolates distribute among the wild-type populations of each molecular type per drug. However, the presence of non-wild-type isolates to more than one antifungal drug, which has been reported from various countries in the region (Table 3 and Table 4), emphasizes the importance of carrying out antifungal susceptibility testing. Amphotericin-B and fluconazole non-wild-type VNI isolates, as well as fluconazole non-wild-type VGII isolates, are of concern, not only because these molecular types are the most common in the region, but also because these antifungals are the drugs of choice for the induction phase of the therapy in most Latin American countries [1]. Susceptibility profiles, although they are not routinely determined in cryptococcal isolates in the region, can be utilized to choose substitute treatments in patients failing to respond to first-line therapy [3,161]. For instance, voriconazole, posaconazole, and isavuconazole, which have shown to have a better in vitro activity against *C. gattii* than fluconazole, could represent valuable alternatives for the treatment of patients with fluconazole-resistant strains [161]. Even though there are no data on clinical breakpoints, the determination of MIC values is also recommended to monitor epidemiological trends [27,28]. As such, further research on antifungal susceptibility in *C. neoformans* and *C. gattii* species complexes is necessary to establish the relationship between MIC, drug dosing, and clinical outcome.

## 5. Conclusions

The increasing application of genotyping methods to study the etiological agents of cryptococcosis in Latin America has generated a large amount of data that have greatly contributed to the global study of the members of the *C. neoformans*/*C. gattii* species complexes, in order to discover their population structure; to recognize their great inter- and intra-species genetic diversity; to understand how these pathogenic yeasts are spreading around the world; and to better define the disease aspects of cryptococcosis, an important mycosis in the region. These results indicate that the identification of cryptococcal species, genotypes, and region of origin may be important when deciding on treatment options for cryptococcosis and to forecast the expansion of the molecular types/species complexes and possible reasons for their emergence and expansion in other regions of the world. Constant clinical and veterinary surveillance, and additional environmental sampling, together with the application of more advanced molecular techniques, such as WGS of a larger set of isolates, are needed to further expand our knowledge on the correlation of the genomic features of the cryptococcal species with their complex biological traits that make them successful pathogens.

## Figures and Tables

**Table 1:** Distribution of the major molecular types of *Cryptococcus neoformans* and *Cryptococcus gattii* species complexes isolates reported from Latin American countries.

			*C. neoformans* Species Complex				*C. gattii* Species Complex				
Country [Ref]	*n*	Source	VNI	VNII	VNIII	VNIV	VGI	VGII	VGIII	VGIV	Total
Brazil [18,20,36,37,38,39,40,41,42,43,44,45,46,47,48,49,50,51,52,53,54,55,56,57,58,59,60,61,62,63,64,65,66,67,68,69,70,71,72,73,74,75,76,77,78,79,80,81,82,83,84,85,86,87,88,89,90,91,92]	2762 ^1^	Cli	1537 ^2^	91	-	2	21	409 ^2,3^	18	-	2078
		Env	365	16	1	23	7	242 ^3^	-	-	654
		Vet	3	-	-	-	-	14	-	-	17
Colombia [18,32,93,94,95,96,97,98,99,100,101,102,103,104,105,106,107,108,109]	1436	Cli	396 ^3^	19 ^3^	-	6	12	64 ^2,3^	40	2	539
		Env	686	6	-	-	4 ^2^	107 ^2^	83	11	897
Argentina [18,20,110,111,112,113,114,115,116,117,118,119]	644	Cli	532	15	31	6	6	2	2 ^3^	-	600
		Env	19	-	-	-	23		2	-	44
Mexico [18,32,120,121]	321	Cli	209	21	12	7	11	5	26 ^2^	7	298
		Env	18	-	-	-	-	-	5	-	23
Cuba [17,35,122,123,124]	247	Cli	141	-	-	36	-	-	1	-	178
		Env	68	-	-	-	-	-	-	-	68
		Vet	-	-	-	-	1	-	-	-	1
Venezuela [18,32,125,126]	101	Cli	70	10	1	-	5	12	3	-	101
Peru [18,127,128]	85	Cli	64	16	2	2	1	-	-	-	85
Costa Rica [129]	36	Cli	22	11	-	-	2	-	-	1	36
Ecuador [130,131]	28	Cli	27	-	-	-	1	-	-	-	28
Chile [18,125,132,133]	20	Cli	4	3	3	5	-	-	-	-	15
		Env	4	-	-	-	-	-	-	-	4
		Vet	-	-	-	-	-	-	1	-	1
Guatemala [18]	15	Cli	14	-	-	-	-	-	1	-	15
Bolivia [134]	1	Cli	-	-	-	-	-	-	1	-	1
Honduras [15]	1	Cli	-	-	-	-	1	-	-	-	1
Paraguay [32]	1	Cli	-	-	-	-	-	-	1	-	1
Uruguay [135]	1	Env	-	-	-	-	-	1	-	-	1
		Cli	3022	186	49	64	60	492	93	10	3976
		Env	1160	22	1	23	34	350	90	11	1691
		Vet	3	-	-	-	1	14	1	-	19
		**Total**	**4185**	**208**	**50**	**87**	**95**	**856**	**184**	**21**	**5686**

^1^ Six clinical and seven environmental VNB isolates are included. ^2^ Mating type **a** was reported among the isolates. ^3^ Pediatric cases were reported among the clinical isolates. Cli: clinical; Env: environmental; Vet: veterinary.

**Table 2:** Genotypic diversity of the *Cryptococcus neoformans* and *Cryptococcus gattii* species complexes populations from Latin America, according to multilocus sequence typing (MLST).

		Population Group			
Species Complex	MT	Clinical	Environmental	Veterinary	Total
*C. neoformans*	VNI	262	76	-	344 ^2^
	VNB	-	5	-	5
	VNII	12	-	-	12
	VNIV	6	-	-	6
	*n* of isolates	280	81	-	367 ^2^
	*n* of STs	34	16	-	41
	*D* ^1^	0.6149	0.7247	-	0.7104
*C. gattii*	VGI	28	2	1	31
	VGII	207	82	10	299
	VGIII	37	32	-	69
	VGIV	1	-	-	1
	*n* of isolates	273	116	11	400
	*n* of STs	125	116	8	149
	*D* ^1^	0.9806	0.9195	0.9273	0.9755

^1^*D*: Simpsons diversity index. ^2^ Six *C. neoformans* isolates were from unknown source. MT: molecular type.

**Table 3:** Minimum inhibitory concentrations of amphotericin-B and 5-fluorocytosine for *Cryptococcus neoformans* and *Cryptococcus gattii* species complexes isolates from Latin America, according to the molecular type. Non-wild-type minimum inhibitory concentrations are underlined.

		Amphotericin-B (µg/mL)		5-Fluorocytosine (µg/mL)		
MT	*n*	Range	GM	Range	GM	Ref.
VNI	99	0.016–0.125	0.099	0.25–8	2.519	[77] ^1^
	26	0.03–0.25	0.060			[79] ^1^
	18	0.03–1	-	0.125–4	-	[89] ^1^
	75	0.03–2	0.348			[116] ^1^
	19	0.125–0.25	-	0.5–8	-	[17] ^1^
	7	0.125–0.25	-			[65] ^2^
	1	0.125	-			[72] ^2^
	17	0.5–1	0.670	0.5–8	2.770	[58] ^1^
	1	0.5	-	2	-	[91] ^5^
VNII	2	0.12	0.120			[116] ^1^
VNIII	4	0.06–0.5	0.173			[116] ^1^
VGI	1	0.06	-	8	-	[60] ^1^
	4	0.125–0.5	0.290			[54] ^3^
	1	0.125	-	8	-	[123] ^1^
	1	0.125	-	0.5	-	[124] ^1^
VGII	4	0.03–0.125	0.060			[79] ^1^
	7	0.03–0.25	-			[90] ^1^
	8	0.03–0.5	0.105	0.5–2	0.771	[89] ^1^
	18	0.06–0.25	0.079	1–8	3.700	[66] ^1^
	50	0.125–0.5	0.390			[54] ^3^
	2	0.125	-	2–4	-	[81] ^1^
	1	0.19	-			[65] ^2^
	10	0.5–1	0.710	1–16	4.92	[58] ^1^
	1	0.5	-	8	-	[59] ^1^
VGIII	54	0.125–2	0.030	0.5–8	2.095	[32] ^4^
	1	0.125	-	4	-	[134] ^1^
	2	0.25	-			[117] ^3^

Methodology to determine antifungal susceptibility: ^1^ M27-A3 guidelines of the Clinical and Laboratory Standards Institute (CLSI), ^2^ E-test strips, ^3^ European Committee on Antimicrobial Susceptibility Testing-Subcommittee on Antifungal Susceptibility Testing (AFST-EUCAST) method E.Def.7.2., ^4^ Sensititre YeastOne plates, and ^5^ Vitek-2. MT: molecular type. *n*: number of studied isolates. GM: geometric mean.

**Table 4:** Minimum inhibitory concentrations of azoles for *Cryptococcus neoformans* and *Cryptococcus gattii* species complexes isolates from Latin America, according to the molecular type. Non-wild-type minimum inhibitory concentrations are underlined.

		Fluconazole (µg/mL)		Itraconazole (µg/mL)		Voriconazole (µg/mL)		Posaconazole (µg/mL)		
MT	*n*	Range	GM	Range	GM	Range	GM	Range	GM	Ref.
VNI	99	0.125–8	0.521	<0.016–0.25	0.026	<0.016–0.125	0.022	<0.016–0.125	0.027	[77] ^1^
	75	0.125–32	2.971							[116] ^1^
	19	0.25–8	-	<0.016–0.5	-	<0.016–0.25	-	0.016–0.125	-	[17] ^1^
	51	0.25–16	7.22							[85] ^1^
	18	0.5–16	-	0.06–0.25	-	0.03–1	-			[89] ^1^
	17	1–16	4.34	0.03–0.25	0.09	0.06–0.5	0.28			[58] ^1^
	1	1	-							[91] ^6^
	26	2–8	4.57	0.03–0.25	0.07					[79] ^1^
	7	2–48	-	0.125–0.5	-					[65] ^3^
	19	4–>64	-							[63] ^2^
	1	32	-							[72] ^3^
VNB	6	4–8	6.86							[85] ^1^
VNII	2	1–2	1.414							[116] ^1^
	14	1–8	2.5							[85] ^1^
VNIII	4	2–4	2.378							[116] ^1^
VNIV	36	0.125–64	-							[35] ^4^
VGI	1	2	-							[123] ^1^
	1	2	-	0.125	-	0.063	-	0.125	-	[124] ^1^
	4	4–8	5.3							[54] ^2^
	1	8	-	0.25	-	0.5	-			[60] ^1^
VGII	18	0.5–16	2.0785	0.031–0.25	0.0994	0.031–0.25	0.0853	0.031–0.25	0.0853	[66] ^1^
	8	0.5–16	4	0.125–0.25	0.1777	0.0031–1	0.148			[89] ^1^
	7	1–8	-	0.03–0.125	-					[90] ^1^
	10	1–16	7.46	0.03–0.5	0.22	0.06–0.5	0.28			[58] ^1^
	50	1–64	12.2							[54] ^2^
	2	4–16	-	1	-	0.12–1	-			[81] ^1^
	10	4–64	25.82							[85] ^1^
	4	8–32	19	0.125–0.5	0.29					[79] ^1^
	1	8	-	0.25	-	0.5	-			[59] ^1^
	1	24	-	0.5	-					[65] ^3^
VGIII	54	1–128	8.239	<0.015–0.125	0.061	<0.008–1	0.033	0.015–0.25	0.057	[32] ^5^
	2	4–16	-	0.125	-	0.06–0.125	-			[117] ^2^
	1	32	-	0.25	-	0.25	-	0.5	-	[134] ^1^

Methodology to determine antifungal susceptibility: ^1^ M27-A3 guidelines of the CLSI, ^2^ AFST-EUCAST method E.Def.7.2., ^3^ E-test strips, ^4^ Casitone broth microdilution method, ^5^ Sensititre YeastOne plates, and ^6^ Vitek-2. MT: molecular type. *n*: number of studied isolates. GM: geometric mean.

## Data Availability

The data supporting this review are from previously reported studies and datasets, which have been cited. The processed data are available from the corresponding author upon request.

## Supplementary Materials

The following are available online at https://www.mdpi.com/article/10.3390/jof7040282/s1, Table S1: Data on the epidemiological cut-off values (ECVs) >95% per drug and molecular type as reported elsewhere.
