# Supplementary material for: Cryptococcus neoformans and Cryptococcus gattii Species Complexes in Latin America: A Map of Molecular Types, Genotypic Diversity, and Antifungal Susceptibility as Reported by the Latin American Cryptococcal Study Group
Source: J Fungi (Basel). 2021 Apr 9;7(4):282. doi: 10.3390/jof7040282 (PMC8069395; doi:10.3390/jof7040282)
Supplement: Supplementary file 1 [file jof-07-00282-s001.pdf]

Supp. Table 1. Data on the epidemiological cut-off values (ECVs) >95% per drug and molecular type as reported elsewhere [25, 26]. When a MIC is lower than or equal to the ECV, the isolates are likely from the wild type distribution of their molecular type for a particular antifungal drug, whereas if the MIC is greater than the ECV the isolates are from a non-wild type distribution and are likely to have an acquired form of resistance.

MT	Amphotericin-B	5-fluorocytosine	Fluconazole	Itraconazole	Voriconazole	Posaconazole
VNI	0.5 µg/ml	8 µg/ml	8 µg/ml	0.25 µg/ml	0.25 µg/ml	0.25 µg/ml
VGI	0.5 µg/ml	4 µg/ml	8 µg/ml	0.5 µg/ml	0.5 µg/ml	0.5 µg/ml
VGII	1 µg/ml	16 µg/ml	32 µg/ml	0.5 µg/ml	0.25 µg/ml	ND
VGIII	ND	ND	8 µg/ml	0.5 µg/ml	ND	ND

ND: Not determined
